# Supplementary material for: PXO_RS20535, Encoding a Novel Response Regulator, Is Required for Chemotactic Motility, Biofilm Formation, and Tolerance to Oxidative Stress in Xanthomonas oryzae pv. oryzae
Source: Pathogens. 2020 Nov 17;9(11):956. doi: 10.3390/pathogens9110956 (PMC7698356; doi:10.3390/pathogens9110956)
Supplement: Supplementary file 1 [file pathogens-09-00956-s001.zip › Supplementary materials for Antar_et al_RR35.docx]

**Supplementary materials for**

*PXO_RS20535,* encoding a novel response regulator, is required for chemotactic motility, biofilm formation, and tolerance to oxidative stress in *Xanthomonas oryzae* pv. *oryzae*

Abdulwahab Antar ^1,2^ Mi-Ae Lee ^1,2^, Youngchul Yoo ^1,2^, Man-Ho Cho^1^ and Sang-Won Lee ^1,2,^*

^1^ Department of Genetic Engineering and Graduate School of Biotechnology, Kyung Hee University, Yongin, South Korea; [abulwahhab@khu.ac.kr](mailto:abulwahhab@khu.ac.kr) (A.A.); [malee0504@khu.ac.kr](mailto:malee0504@khu.ac.kr) (M.L.); [yooyc@khu.ac.kr](mailto:yooyc@khu.ac.kr) (Y.Y.)

^2^ Crop Biotech Institute, Kyung Hee University, Yongin, South Korea

***** Correspondence: [swlee6803@khu.ac.kr](mailto:swlee6803@khu.ac.kr)

1. Table S1. Bacterial strains and primers used in this study

2. Table S2. Fold changes of transcripts regulated in RR35 from RNA sequencing analysis.

3. Supplementary Figure 1

4. Supplementary Figure 2

5. Supplementary Figure 3

**Table S1. Bacterial strains and primers used in this study**

| **Strain or primer** | **Description or sequence** | **Reference or source**  **s** |
| --- | --- | --- |
| ***Xanthomonas oryzae* pv. *oryzae*** | |  |
| PXO99A | Wild-type, Philippine race 6 strain; Cp^r^ | Lab collection |
| RR35 | *PXO_ RS20535* knockout mutant with kanamycin  insertion at nucleotide 23; Cp^r^ and Km^r^ | Han et al., 2019 |
| cRR35 | RR35 complemented with pBBR1-MCS5 inserted  by 6XHis-*PXO_RS20535*; Cp^r^, Km^r^ and Gm^r^ | Han et al., 2019 |
| **Primers** |  |  |
| 16S rRNA |  |  |
| RS22200-F | 5'-TACGTTATCCCCCACGAAAG-3' |  |
| RS22200-R | 5'-GTTTGATCCTGGCTCAGAGT-3' |  |
|  |  |  |
| Biofilm and Motility |  |  |
| RS11980-F | 5'-GACGGTATCGATTTGCGTGG-3' |  |
| RS11980-R | 5'-GCAGCTTCTCCACCAATGTG-3' |  |
| RS12875-F | 5'-GGTGCTCAACCTCACCAAGG-3' |  |
| RS12875-R | 5'-GCGTCGTAACTGCTGCTTTT-3' |  |
| RS12870-F | 5'-TACGTATTTTGCAACGCGCA-3' |  |
| RS12870-R | 5'-AGGTCCAGATGGGTGGGAT-3' |  |
| RS12145-F | 5'-CTGGTGCTGTCGCTGTACTA-3' |  |
| RS12145-R | 5'-CATCGGCCAGTTCGAAGACT-3' |  |
| RS12855-F | 5'-AAGAGGAGTTCGAGGACATGC-3' |  |
| RS12855-R | 5'-CGCATCATCCGCATCAGGT-3' |  |
| RS01540-F | 5'-GGATCAAGCCGCTTTCTTCG-3' |  |
| RS01540-R | 5'-CAACCGATCATTGCACTCGAC-3' |  |
|  |  |  |
| Oxidative stress |  |  |
| RS05465-F | 5'-AATGGCAGTGCAAGCAACG-3' |  |
| RS05465-R | 5'-CCGTATTTGTTGCCGCATGT-3' |  |
| RS17325-F | 5'-GCGCAACAGTCAAGATCGTC-3' |  |
| RS17325-R | 5'-CCGCTTCCGAAGACAGTAGG-3' |  |
| RS22725-F | 5'-GAACGTGGGGAAGTTGTTGC-3' |  |
| RS22725-R | 5'-GTCTTCGTGCGCTTTTCCTC-3'' |  |
| RS22555-F | 5'-CGTTCGGCAAGCAATATCCG-3' |  |
| RS22555-R | 5'-ATGTAGCGTTCGCGTCCATC-3' |  |
|  |  |  |
| T6SS |  |  |
| RS07340-F | 5'-GCGAAAGTGGATATCGGGCG-3' |  |
| RS07340-R | 5'-GCGTGCCGAAGACAGACTGA-3' |  |
| RS07330-F | 5'-GCAGTCGGACTACGTGACGA-3' |  |
| RS07330-R | 5'-TCCTGCGGCTGGAACGAATA-3' |  |
| RS07400-F | 5'-TCCACGGCGAAACCTATGGC-3' |  |
| RS07400-R | 5'-CACCCTGCTCCACCCGATAG-3' |  |
| RS07300-F | 5'-ATCTGGATGCGCAGCAGGTG-3' |  |
| RS07300-R | 5'-ACCTGCAGGCACAGGAACTG-3' |  |
| RS17855-F | 5'-GATAAACCGGCCTGGGTCAA-3' |  |
| RS17855-R | 5'-AGTGCCTGAATGCGGGAAAA-3' |  |
| RS07355-F | 5'-CCTGTGCAAGGACCCGGAAT -3' |  |
| RS07355-R | 5'-TGTCCGAGCATGGCGTTACT-3' |  |
| RS17625-F | 5'-GACCTACGACGTGGAAAAGG-3' |  |
| RS17625-R | 5'-AGGTCGACGTTGACGAATTT-3' |  |
|  |  |  |
| RS01535-F | 5'-GATAAAGGCGGCCGAATTGC -3' |  |
| RS01535-R | 5'-CACGTTGGCGGCAAGATTAC -3' |  |
| RS19530-F | 5'-GATAAAGGCGGCCGAATTGC -3' |  |
| RS19530-R | 5'-CACGTTGGCGGCAAGATTAC -3' |  |
| RS06115-F | 5'-TATCAACGCCGATGTGTTGG-3' |  |
| RS06115-R | 5'-GGAGTCTTTCTCAGCCTGC-3' |  |
| RS17955-F | 5'-ATTTCTATGCCATCACCGGC-3' |  |
| RS17955-R | 5'-GGTGTTCTCGAAGCTGACTT-3' |  |
| RS17835-F | 5'-CTGCCGGTCGTATTGATCG-3' |  |
| RS17835-R | 5'-GCCAACCAGCGTTCGAC-3' |  |
| RS09085-F | 5'-AGATCAATGCGTTCGCTACC-3' |  |
| RS09085-R | 5'-GACCATGTCGCCATTAGCAA-3' |  |
| RS13625-F | 5'-ACAATTGCTCAAGGTTGCCG-3' |  |
| RS13625-R | 5'-TAGCCCTTCTGGAACACCTG-3' |  |
| RS23120-F | 5'-CCTATGTCGAAACGCACGA-3' |  |
| RS23120-R | 5'-AGATGTTCCAGCGCGATCT-3' |  |
| RS11120-F | 5'-GAGACTGATCCAACCCTTG-3' |  |
| RS11120-R | 5'-CATGATGGTGGGGATATTGG-3' |  |
| RS07405-F | 5'-GGGCCTGCTGAGTGATGTCT-3' |  |
| RS07405-R | 5'-ACCCAATCGTCGGCGAGAAA-3' |  |
| RS06900-F | 5'-ATGGTACGGGCCTAGTCA -3' |  |
| RS06900-R | 5'-CAGGTACTTGGTGAGGATGG -3' |  |
| RS16940-F | 5'-GTCGGTGGATTGAACTGGGG -3' |  |
| RS16940-R | 5'-CTGGTGTGATGCTCGGCAA -3' |  |
| RS05075-F | 5'-AGATCCAGATCATGACCGC-3' |  |
| RS05075-R | 5'-CAGAATTAACTTGTCGTTGCTG-3' |  |
| RS02070-F | 5'-TTTTCCAGGCACGCACCTT-3' |  |
| RS02070-R | 5'-TCAGTTCGATCAGCGGCAAT-3' |  |
| RS23210-F | 5'-CCTATGTCGAAACGCACGA-3' |  |
| RS23210-R | 5'-ACATGTTCCAGCGCGATCT-3' |  |
| RS01530-F | 5'-CACGCATCGACACCTATCCG-3' |  |
| RS01530-R | 5'-GAAGGCTTCGGCCTGAAATG-3' |  |
| RS14970-F | 5'-CGAATTGTATTGATCGAGGAC-3' |  |
| RS14970-R | 5'-CCATCAACACAAGATCGGG-3' |  |
| RS18925-F | 5'-GACGACTATCTGATCAAGCC-3' |  |
| RS18925-R | 5'-AGCGTATCCAGGTTGTATTC-3' |  |
|  |  |  |

Cp^r^, Km^r^ and Gm^r^ indicate resistance to cephalexin, kanamycin, and gentamycin, respectively

Supplementary Figure 1


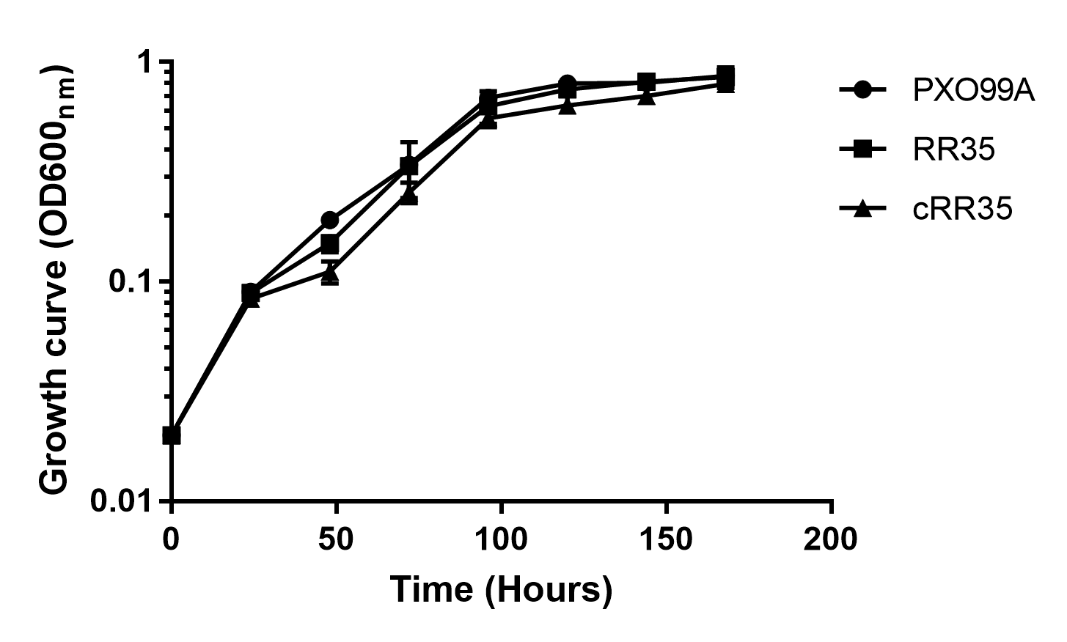


**Supplementary Figure 1.** Growth of PXO99A, RR35, and cRR35 populations in XOM2. Bacterial strains were cultured in 100ml XOM2 media with shaking. Samples were measured at the same time for 7 days using a spectrophotometer. The experiments were repeated four times. The error bars are standard deviations of two broth media for each strain.

Supplementary Figure 2


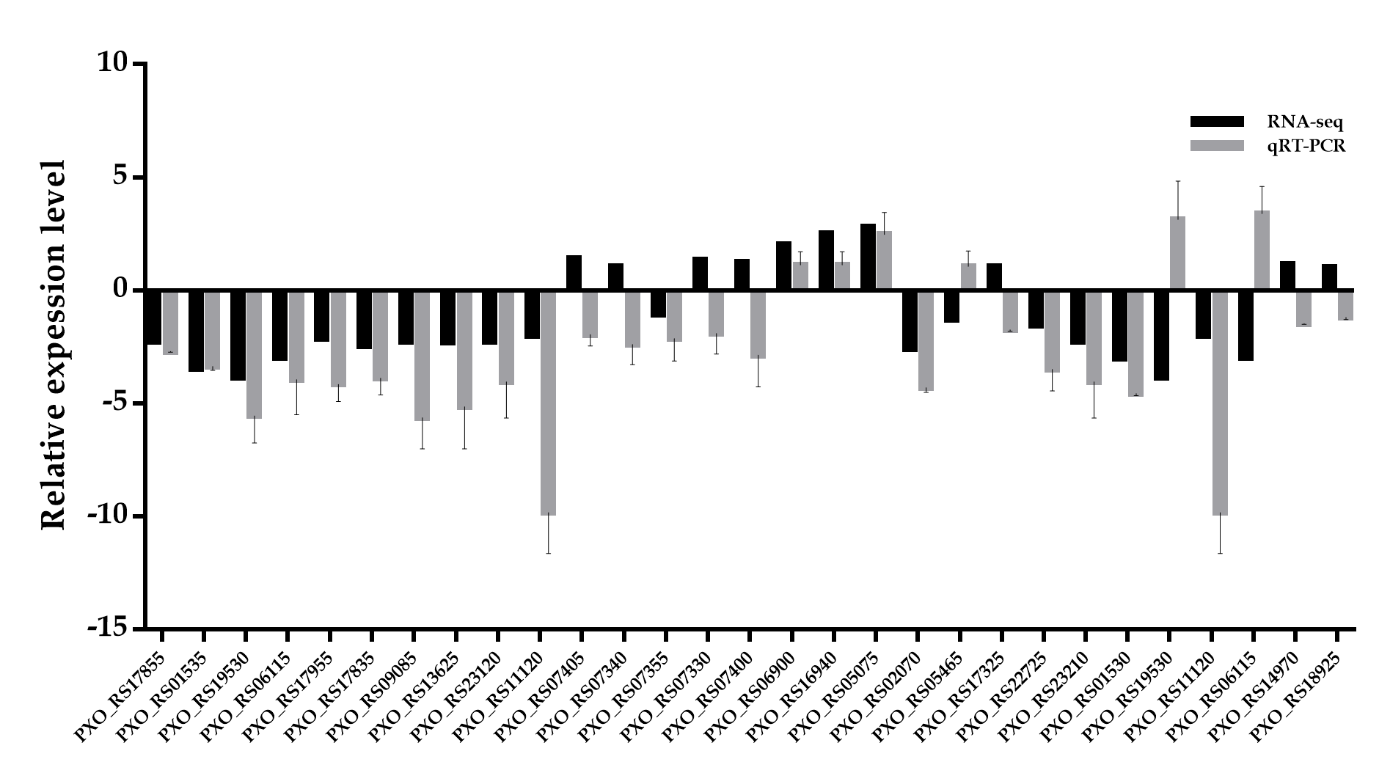


**Supplementary Figure 2.** Differentially expressed genes (DEGs) validated by qRT-PCR. Comparison of gene expression between RNA-sequencing data and qRT-PCR validation results. The X-axis shows 29 genes involved in virulence factors of pathogenic bacteria. The y-axis shows fold-change expression values determined by RNA-sequencing data and qRT-PCR.

Supplementary Figure 3


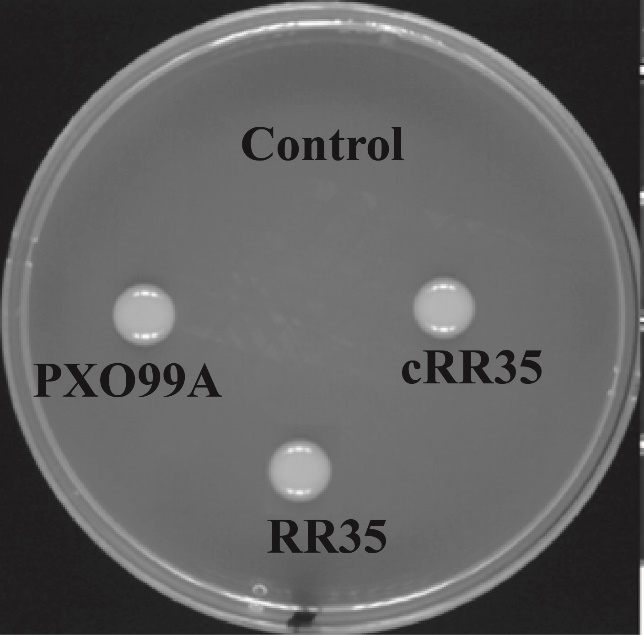


**Supplementary Figure 3.** Growth of PXO99A, RR35, and cRR35 on the XOM2 agar plate. Bacterial strains were dropped onto 1.5 % XOM2 agar plates and incubated at 28 °C for 7 days. This growth control test was repeated three times, and results from one experiment are shown.
